# The Beneficial Role of Exercise Training for Myocardial Infarction Treatment in Elderly

**DOI:** 10.3389/fphys.2020.00270

**Published:** 2020-04-24

**Authors:** Ying Xing, Si-Dong Yang, Man-Man Wang, Ya-Shuo Feng, Fang Dong, Feng Zhang

**Affiliations:** ^1^Department of Rehabilitation Medicine, The Third Hospital of Hebei Medical University, Shijiazhuang, China; ^2^Australian Institute for Bioengineering and Nanotechnology, The University of Queensland, Brisbane, QLD, Australia; ^3^Department of Orthopedic Surgery, The Third Hospital of Hebei Medical University, Shijiazhuang, China; ^4^Department of Clinical Laboratory Medicine, The Third Hospital of Hebei Medical University, Shijiazhuang, China; ^5^Hebei Provincial Orthopedic Biomechanics Key Laboratory, The Third Hospital of Hebei Medical University, Shijiazhuang, China

**Keywords:** aging, cardio protection, cardiopulmonary rehabilitation, exercise, myocardial infarction

## Abstract

Worldwide, elderly people have a higher prevalence of myocardial infarction (MI), which is associated with body function aging and a sedentary lifestyle. In addition to medication, exercise training is a well-established supplementary method to prevent and treat cardiovascular diseases (CVDs). Substantial evidence has shown the value of different intensity exercise programs in the prevention and treatment of MI, and exercise rehabilitation programs are also applicable to elderly patients with MI. Although exercise rehabilitation programs could significantly improve function, quality of life (QoL), and lower mortality and morbidity for people with MI, such programs are underused because their mechanisms are not accurately elucidated. To promote the application of exercise therapy for MI, this review summarizes the benefits and mechanisms of exercise rehabilitation for post-MI patients and provides rationalized proposals for outpatient cardiac rehabilitation.

## Highlights:

– Exercise therapy contributes to improve behavioral risk factors that may result in MI, promotes exercise capacity, and elevates QoL for MI patients.
– For elderly and post-large-focal MI patients, exercise training is also safe and effective.
– Early exercise training, even short-term exercise, is a safe and feasible way to exert protective effects in post-MI patients.
– In the early stages of MI, moderate-intensity exercise is the best choice to improve the outcomes for MI patients.
– Cardiovascular rehabilitation and interval exercise had unique advantages, which should be recommended for MI patients.

## Introduction

Myocardial infarction (MI) is related to formation of plaques in the inner wall of the artery, which blocks or reduces blood flow to the heart and damages heart muscles because of the lack of oxygen supply (Lu L. et al., 2015). In China, the mortality of acute MI increased 5.6-fold from 1987 to 2014 (Chang et al., 2017). Individuals aged 8,084 years have 190.70 and 220.15 times higher mortality risk of acute MI compared to those aged 1,519 years in Chinese rural and urban populations, respectively (Chang et al., 2017). A total of 2,812 elderly patients followed on 9 years showed that disability in basic strength and mobility increased the year following being diagnosed with MI (Mendes de Leon et al., 2005).

Currently, multiple therapy options, including thrombolytic drugs, percutaneous transluminal coronary angioplasty (PTCA), and coronary artery bypass grafting, are available to treat acute MI in clinic (Kahn et al., 1993; Sorensen and Maeng, 2015; Lhermusier et al., 2019; Song et al., 2019). In addition to medications and surgeries, epidemiological evidence has shown that exercise, such as stair climbing, walking, and sports, is inversely correlated to the mortality of cardiovascular causes (Paffenbarger Jr., Hyde et al., 1986). Thus, exercise is an effective supplementary therapy and usually plays a key role in the process of treatment for patients with acute MI. Exercise training in patients with acute MI can improve work load, functional capacity, test duration, and heart rate response (Andjic et al., 2016), as well as promote the improvement of cardiac pump function – a 34.7 and 32.0% mean rise in ejection fraction and stroke index, respectively (Chursina and Molchanov, 2006).

In this review, we summarize the evidence for the beneficial effect of exercise rehabilitation programs for MI from randomized controlled trails (RCTs), epidemiological reports, meta-analysis and clinical studies, and laboratory experiments so as to extend the application of exercise in the prevention and treatment for MI.

## Exercise and Age-Related MI

**FIGURE 1:**
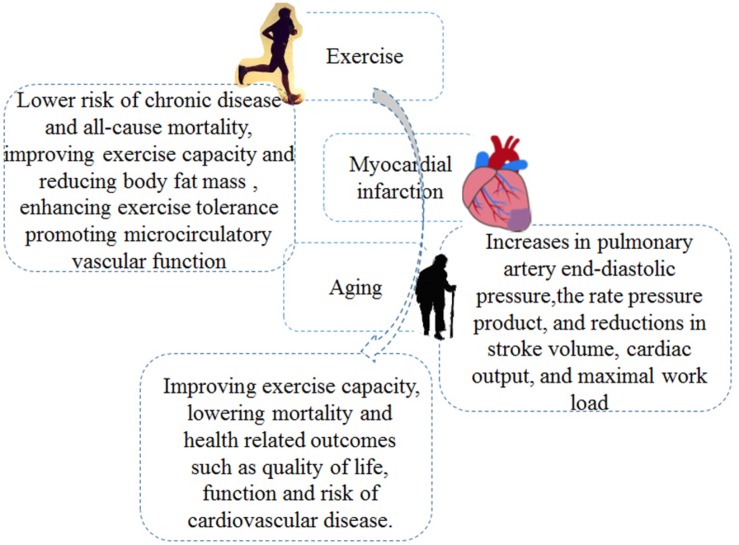
The involved mechanisms for exercise intervention in myocardial infarction (MI) treatment in elderly.

According to epidemiological results, aging will become a main risk factors for CVD after the age of 65 (North and Sinclair, 2012). Aging is independently associated with peak oxygen uptake (VO_2_ peak) and total work capacity (TWC), accounting for nearly 70% of the age-related decay (Marchionni et al., 2000). Controlling life risk factors including physical inactivity and sedentary behaviors might be an effective method to reduce global mortality and morbidity in patients with CVD (Fletcher et al., 2018; Blaum et al., 2019; Lavie et al., 2019). Lifelong (>25 years) exercise may alleviate a sedentary- and aging-induced decrease in systolic longitudinal strain (LS) through improving left ventricular (LV) diastolic filling (Howden et al., 2018). Regular exercise plays an important role in healthy aging and contributes to lower risk of chronic disease and all-cause mortality (Mora and Valencia, 2018; Adams and Linke, 2019). The lowered risk of cardiovascular events in elderly individuals (age, 66.6 ± 2.1 years) was associated with improving exercise capacity (+ 2.0 ml kg^–1^ min^–1^) and reducing body fat mass (–2.3%) (Niederseer et al., 2011). Regular moderate-intensity training (MIT) for 8 weeks enhanced exercise tolerance and promoted microcirculatory vascular function in postmenopausal women (Alkhatib and Klonizakis, 2014). In summary, exercise training could improve the physical function and parameters of MI related to aging, indicating that the elderly should adhere to appropriate physical exercise, which is conducive to heart health (as shown in Figure 1).

## The Benefits of Exercise for MI

Exercise training concerns planned and organized body movement to improve physical capacities; examples include swimming, yoga, aerobic and resistance/strength exercise, and so on (Tulpule and Tulpule, 1980; Ferrera et al., 2014; Moraes-Silva et al., 2017; Ostman et al., 2017). After MI, Exercise training may induce positive effects; improve QoL, metabolic equivalents (METs), circulation function, and heart rate; and lower the risk of chronic disease and all-cause mortality (Greif et al., 1995; Adams et al., 2017; Elshazly et al., 2018; Mora and Valencia, 2018) (as shown in Figure 1).

Exercise training exerted beneficial effects in the process of cardiopulmonary rehabilitation and LV remodeling in the LV dysfunction patients after MI, and the greatest effects were achieved when exercise began at the post-MI acute phase (Zhang et al., 2016). A cross-sectional study of 65 men (60 ± 6 years) found that lifelong exercise training maintained LV systolic function and probably alleviated or minimized the detrimental effects of LV remodeling after MI in veteran athletes (Maessen et al., 2017). LV end diastolic and systolic volumes had significantly decreased in MI patients after 10 weeks of exercise training (Mc et al., 2016). Following acute MI, patients who participated in interval training or MIT for 12 weeks significantly increased their VO_2_ peak (Santi et al., 2018). In summary, exercise training effectively promoted cardiac circulation by improving cardiac performance in MI patients.

Dynamic resistance training may alleviate sympathetic tonus to the heart vessels in rats after MI (Barboza et al., 2016). Resistance exercise training for 3 months reduced the vascular and cardiac sympathetic regulation and increased the parasympathetic regulation so as to improve cardiac autonomic balance in post-MI rats (Grans et al., 2014). The improvement of activated sympathetic drive was associated with elevated NO bioavailability in paraventricular nucleus (PVN) of chronic heart failure rats induced by MI during 3-week progressive treadmill exercise (Sharma et al., 2019). In summary, exercise regulated the autonomic balance of nerves in MI patients, resulting in an improvement of cardiac performance and a reduction in cardiac mortality.

## Different Types of Exercise Programs and MI

### Cardiac Rehabilitation and MI

Exercise-based cardiac rehabilitation (CR) is a multidisciplinary program for individuals after MI to reduce cardiorespiratory fitness (CRF), morbidity and mortality as well as improve QoL and exercise capacity (Franklin et al., 2013; Korzeniowska-Kubacka et al., 2015; Tessitore et al., 2017). It covers 10 domains of cardiac risk factor regulation, including weight management, exercise training, patient assessment, and so on (Costantino et al., 2016; Richardson et al., 2019). The main benefits associated with CR are produced by exercise training (Oldridge, 2012; Lewinter et al., 2015; Anderson et al., 2016).

In a cohort study, 37 patients (mean age, 66 years) with MI underwent a 5-week CR program, and the results indicated that cardiac rehabilitation improved QoL, exercise capacity, and autonomic modulation (Fallavollita et al., 2016). Kim et al. suggested that a 6-week CR exercise program with an intensity of 60–85% heart rate reserve improved cardiopulmonary function in patients with ischemic cardiomyopathy (Kim et al., 2016). Patients with a home-based walking program showed an obvious improvement of functional capacity, increasing their inspiratory muscle endurance (PTHmax) and maximal inspiratory pressure (MIP) in 15 and 60 days following MI (Matos-Garcia et al., 2017). A study of 359 patients with acute MI who underwent a CR program (6-week hospital- or home-based aerobic exercise) suggested that those patients had significant improvements in their resting heart rate, VO_2_ peak, total exercise duration (TED), and METs after cardiac rehabilitation, regardless of obesity (Lim et al., 2016).

After percutaneous intervention, patients with a 4-week outpatient CR program had obvious improvements in their maximum VO_2_ peak and METs (Choe et al., 2018). CR for 3 years showed reduced major adverse cardiovascular events (e.g., MI) compared to those without CR (9.9 vs. 18.3%) (Lee et al., 2019). Moreover, CR contributed to a decrease in all-cause mortality, cardiac mortality, and reinfarction risk (Lawler et al., 2011) and helped to regulate cardiovascular-risk-related factors, such as blood pressure, body weight, smoking, and lipid profile (Lawler et al., 2011).

In summary, as shown in Table 1, not only did CR lower cardiac mortality and improve QoL and exercise capacity, but it also ameliorated cardiovascular risk factors in the basis of multidisciplinary program, which was mainly due to benefits induced by exercise training.

**TABLE 1:** Exercise and MI.

References	Time	Disease	Participation	Outcome
**Cardiac rehabilitation**				
Fallavollita et al. (2016)	5 weeks	MI	37 patients	Improvements in QoL exercise capacity (from 423 ± 94 to 496 ± 13 m) and autonomic modulation
Kim et al. (2016)	6 weeks	Ischemic cardiomyopathy	48 patients	Improving cardiopulmonary function and increasing LVEF.
Matos-Garcia et al. (2017)	2 months	MI	31 patients	Improvement of functional capacity by increasing PTHmax and MIP
Lim et al. (2016)	6 weeks	MI	359 patients	Improvements in HRrest, VO_2_ peak, TED and METs
Choe et al. (2018)	4 weeks	MI	66 patients	Improvements in VO_2_ peak and METs.
Lee et al. (2019)	3 years	MI	265 patients	Reduced major adverse cardiovascular events (e.g., MI) than those without CR (9.9% vs. 18.3%).
**Moderate-intensity exercise**				
Cai et al. (2018)	Moderated-intensity exercise	MI	10 rats	Suppress skeletal muscle atrophy
Aronov et al. (2009)	Moderate-intensity exercise	Ischemic heart disease.	197 patients	Increases of efficiency of cardiac work and work performed volume (+ 74.3%), prolongation of exercise time (+ 31.7%), structural functional improvement of heart
Alkhatib and Klonizakis (2014)	Moderated-intensity exercise	Sedentary postmenopausal participants	15 patients	Improvement of microcirculatory perfusion cardiorespiratory capacity
Aronov et al. (2009)	Moderated-intensity exercise	Acute coronary events	188 patients	Lowering atherogenic index, total cholesterol and body mass index
Xu et al. (2018)	Moderate exercise	MI	10 rats	Promoting α-myosin heavy chain (α-MHC) expression and myocardial contractile function, and improve prognosis.
**High- and low-intensity exercise**				
Wisloff et al. (2006)	High-intensity exercise	Cardiovascular disease	27,143 men and 28,929 women	Lower the cardiovascular death risk
Ades et al. (1996)	High-intensity exercise	Elderly patients with coronary bypass surgery or myocardial infarction	60 patients	16 and 20% increase in peak aerobic capacity and increased the difference of arteriovenous oxygen at peak exercise
Kemmler et al. (2013)	Intense multipurpose exercise	Osteopenic Caucasian females	137 patients	Improve metabolic and lower cardiac risk
Al’khimovich et al. (1985)	Intensive exercise	large-focal myocardial infarction	21 patients	Improved myocardial functional potentials, better physical stress tolerance, better psychological outlook and smaller pulmonary venous congestion
Hua et al. (2009)	Low-intensity exercise	Mildly hypertensive men and women	20 patients	Improved VO_2_ peak
Worcester et al. (1993)	Low-intensity exercise	MI	224 patients	Improvement of QoL

### Physical Activity and MI

Physical activity (PA) is a crucial preventive measure against CVD (Jefferis et al., 2019), which is recognized as part of occupation, active transportation, leisure, and daily living, such as walking for several minutes in the park and chatting with a friend, and the leg muscles voluntarily contract and the energy expenditure ascends exponentially from baseline levels (Moraes-Silva et al., 2017).

Physical activity in patients with acute MI, even at a low intensity, can play an important role in improving health-related QoL (Lovlien et al., 2017). Renninger et al. (2018) suggested that leisure-time physical activity was an independent factor in association with risk of MI, and it might reduce the risk of MI-related excess bodyweight. In a prospective study on postmenopausal MI survivors, patients with increased physical activity following a first MI showed a reduced risk of all-cause mortality than patients with low physical activity (Gorczyca et al., 2017). Elderly patients (age > 65 years) who underwent the highest level activity had a lower mortality from CVD than those who underwent the lowest level activity (Park et al., 2012). After MI, elderly patients with pre-infarction angina who participated in a high level of physical activity had a lower in-hospital mortality compared to those without pre-infarction angina (Abete et al., 2001). High level of physical activity could restore the protective effect of pre-infarction angina on lower in-hospital mortality in elderly patients after MI (Abete et al., 2001).

In summary, physical activity can play a crucial role in reducing mortality of CVD in post-MI patients. Therefore, daily physical activity is important to MI patients, especially for elderly patients with low levels of physical activity. Well-planned and high-level physical activity can also help elderly people reduce the mortality risks associated with CVD.

### Moderate-Intensity Exercise and MI

Moderate-intensity continuous training (MICT) is one of the best choices for exercise rehabilitation during the early stages following MI (Cai et al., 2018). Such physical training showed sufficient efficacy in the physical capacity of 197 patients during the early stage of ischemic heart disease, including an increase in the efficiency of cardiac work and work performed volume (+74.3%, *p* < 0.001) as well as the prolongation of exercise time (+ 31.7%, *p* < 0.001) (Aronov et al., 2009). Microcirculatory perfusion cardiorespiratory capacity also improved in sedentary postmenopausal participants after MIT for 8 weeks as evidenced by this ventilator threshold: 11.5 ± 2.1 vs. 14.0 ± 3.0 ml kg^–1^ min^–1^, *p* < 0.05 (Alkhatib and Klonizakis, 2014).

MIT was helped reduce atrioventricular (AV) block cycle length, AV intervals, sinus cycle length, and ventricular effective refractory period (Kannankeril and Goldberger, 2002); it also led to a significant structural functional improvement of the heart via increasing ejection fraction (7.2%) and LV stroke volume (4.5%) while reducing LV volume (2.5%) and systolic LV volume (8.1%) in individuals with ischemic heart disease (Aronov et al., 2009).

### High-Intensity Exercise

The benefits of high-intensity training (HIT) was twice as good as MIT through analyzing VO_2_ peak in healthy subjects and patients with heart disease (Kemi and Wisloff, 2010). Participation in exercise once a week could lower the risk of cardiovascular death both in women and men. The risk reduction induced by exercise promoted with age for men (Wisloff et al., 2006). A 12-year-long clinical study reported that subjects with an intense multipurpose exercise program effectively improved metabolic parameters and lowered cardiac risk in postmenopausal women as compared to those with habitual physical activity (Kemmler et al., 2013).

In addition, high-intensity interval training (HIIT) was considered as a beneficial and feasible supplementary therapy in international clinical-based exercise guidelines to MICT (Kim et al., 2015; Taylor et al., 2019). Patients with MI who participated in HIIT had greater decreases in fat mass, body fat percentage, waist circumference, abdominal fat percentage, low-density lipoprotein cholesterol, total cholesterol, triglycerides, and greater improvements in body composition and metabolic syndrome as compared to MICT (Dun et al., 2019a, b). HIIT was also superior to MICT in decreasing oxidative stress, improving glucolipid metabolism, and enhancing exercise capability and cardiac function in post-MI rats (Lu K. et al., 2015).

### Low-Intensity Exercise and MI

Long-term (4 months) low-intensity training (LIT) mitigated the enhancement of myocardial type I and III collagen and lysyl oxidase gene expression in LV (Pagan et al., 2015). In a randomized controlled trial lasting 12 weeks, patients with CVD received LIT or HIT, and the significant improvement in VO_2_ peak had no significant difference (Hua et al., 2009). The improvement of QoL provided by LIT for 11 weeks was similar to HIT during the early stages of acute MI (Worcester et al., 1993).

In summary, as shown in Table 1, well-planned HIT may have better effects than MIT and LIT, while LIT may be safer compared to MIT and HIT. Moreover, MIT was both safe and effective for MI patients; it lowers possible risks as compared to HIT and had better effects as compared to LIT. Therefore, MIT was most commonly used in clinics.

### Interval Exercise and MI

In a randomized control study on patients with MI, both aerobic interval training and usual care rehabilitation increased serum adiponectin, improved endothelial function and QoL, and decreased resting heart rate and serum ferritin; only aerobic interval training, however, increased the level of high-density lipoprotein cholesterol, which could exert benefits for patients (Moholdt et al., 2012). Interval training also had a more beneficial effect in improving VO_2_ peak from 31.6 ± 5.8 to 36.2 ± 8.6ml kg^–1^ min^–1^ as compared to the usual care rehabilitation, which was from 32.2 ± 6.7 to 34.7 ± 7.9ml kg^–1^ min^–1^ (Moholdt et al., 2012). After 12 weeks of interval training, the VO_2_ peak had increase from 19.2 ± 5.1 to 21.9 ± 5.6 ml kg^–1^ min^–1^ in 31 patients (55.1 ± 8.9 years) with MI in the anterior wall (Santi et al., 2018). Thus, participation in interval exercise had unique advantages as compared to other types of exercise training for MI patients, which needs further research in the future.

### Resistance and Aerobic Exercise and MI

Resistance exercise (RT) with weight training machines, even one time or < 1 h/week, is related to lower risks of CVD and global mortality (Liu et al., 2019). In animal experiment, LM et al. indicated that aerobic exercise and dynamic RT might decrease pro-inflammatory cytokine level and alleviate sympathetic tonus to the vessels and heart in rats after MI (Barboza et al., 2016). A meta-analysis on 35 randomized controlled trials showed that isolated progressive resistance training exerted beneficial effects in lower (standardized MD, 0.57; 95% CI, –0.17 to –0.96) and upper [1.43 (0.73–2.13)] body strength. In addition, progressive resistance training plus aerobic training was more effective in both strength and fitness than aerobic training alone (Ostman et al., 2017). Twelve-month resistance in combination with aerobic exercise at a 2 days/week frequency may improve muscle strength and cardiorespiratory fitness in all age groups (Ciolac et al., 2014).

### Swimming and MI

Swimming is a popular recreational activity and unique exercise form, regarded as an effective exercise to maintain and improve CRF (Lazar et al., 2013). In animal experiment, 3-week swimming training may alleviate acute-MI-caused acute cardiac damage by elevating the early adaptive altering of mitochondrial biogenesis and improving myocardial energy metabolism (Tao et al., 2015).

### Yoga and MI

Yoga-based lifestyle intervention may significantly decrease estimated 10-year cardiovascular disease (CVD) risk and Framingham Risk Score (FRS), so as to obviously lower CVD risk (Yadav et al., 2017). There was also an obvious shift from sympathovagal balance toward parasympathetic predominance and increase in overall heart radio variability in MI patients with optimally medication treatment (Christa et al., 2019).

## Different Exercise Time and MI

### The Benefits of Exercise Intervention Before MI

In one study with animals, exercise pretreatment preserved cardiomyocyte contractile and morphological properties, which played a crucial role in cardioprotection against cardiac structural deterioration and dysfunction caused by MI (Bozi et al., 2013). Exercise pretreatment could also reduce collagen accumulation, thicken infarcted wall, alleviate MI volume, improve muscle strength, enhance responsiveness to calcium, and preserve cardiac myocyte shortening; it could also improve the maximum relengthening and shortening velocities in infarcted hearts of rats (Bozi et al., 2013; Ciolac et al., 2014). There was a close relationship between cardio protection against myocardial injury induced by exercise pretreatment and cardiac natriuretic peptide receptor B (NPR-B) and C-type natriuretic peptide (CNP) (Lu and Pan, 2017). In summary, exercise before MI could benefit the recovery process following MI.

### Exercise in the Early Stages of MI

Early exercise programs were beneficial to patients with MI through improving psychological responses to exertion and promoting functional capacity, even short-term exercise training (Williams et al., 1985; Greif et al., 1995). Early exercise training also helped improve exercise tolerance, ventricular remodeling, and autonomic nerve balance in post-MI patients (Batista et al., 2013). However, Batista et al. (2013) demonstrated that delayed exercise may exert better effects than early exercise. Therefore, the reasonable exercise time requires further exploration so as to provide rational advice for MI patients.

## Conclusion

CVD such as MI are associated with poor health behaviors, such as a sedentary lifestyle. Exercise therapy is an effective intervention method to improve behavioral risk factors that may result in MI, promote exercise capacity, and elevate QoL for MI patients. Even low-level physical activity reduced the risk of MI. Therefore, daily physical activity should be recommended to people with or without MI instead of a sedentary lifestyle. For elderly and post-large-focal MI patients, exercise training is also safe and effective, but it should be further confirmed in future research. Early exercise training, even short-term exercise, is also a safe and feasible way to improve functional capacity, exercise tolerance, ventricular remodeling, and autonomic nerve balance in post-MI patients. In the early stages of MI, MIT is the best choice to improve the outcomes for MI patients. In addition, CR programs and interval exercise had unique advantages, which should also be recommended for MI patients. The combination of RT and aerobic exercise is an effective therapy to lower the risk of CVD. The intervention of swimming and yoga can effectively improve sedentary lifestyle, so as to lower the risk of CVD. In conclusion, exercise training is an effective and reliable alternative treatment for MI patients in the basis of medication and surgery therapy, as it has fewer side effects and more long-lasting benefits. This type of treatment should be standardized and widely applied in clinics to help MI patients all over the world.

## Author Contributions

YX, S-DY, and FZ had the idea for the article and drafted and critically revised the work. M-MW, Y-SF, and FD performed the literature search and data analysis.

## Conflict of Interest

The authors declare that the research was conducted in the absence of any commercial or financial relationships that could be construed as a potential conflict of interest.
